# Short Tandem Repeat (STR) Somatic Mutation in Non-Melanoma Skin Cancer (NMSC): Association with Transcriptomic Profile and Potential Implications for Therapy

**DOI:** 10.3390/cancers17101669

**Published:** 2025-05-15

**Authors:** Muhammad G. Kibriya, Armando Almazan, Maria Argos, Tariqul Islam, Christopher R. Shea, Habibul Ahsan, Farzana Jasmine

**Affiliations:** 1 Institute for Population and Precision Health (IPPH), University of Chicago, Chicago, IL 60637, USA; armando.almazan@bsd.uchicago.edu (A.A.); habib@uchicago.edu (H.A.); farzana@uchicago.edu (F.J.); 2 Department of Public Health Sciences, Biological Sciences Division, University of Chicago, Chicago, IL 60637, USA; 3 Department of Environmental Health, School of Public Health, Boston University, Boston, MA 02118, USA; argos@bu.edu; 4 UChicago Research Bangladesh (URB), University of Chicago, Dhaka 1230, Bangladesh; 5 Division of Dermatology, Department of Medicine, University of Chicago, Chicago, IL 60637, USA; cshea@bsd.uchicago.edu

**Keywords:** non-melanoma skin cancer, basal cell carcinoma, somatic mutation, short tandem repeat, homopolymeric region, immune checkpoint inhibitors, hedgehog signaling, Notch signaling, anti-VEGF, proteasome inhibitor

## Abstract

Background: Studies on somatic mutations in cancer typically report single-nucleotide variants in coding regions, while mutations in short tandem repeats (STRs) are usually overlooked. Homopolymeric regions, a subset of STRs, are stretches of DNA where only a single nucleotide is repeated multiple times (e.g., AAAAA or TTTTT). Only recently have mutations in such STR regions been seen in colorectal cancer, where microsatellite instability (MSI) is common. In non-melanoma skin cancer (NMSC), MSI is rare. In this study, we focus on somatic mutations in such homopolymeric regions in NMSC and their functional implications. Methods: We performed targeted DNA sequencing (paired tissue and blood from the same individual), using more than 400 cancer-related genes from 32 NMSC patients as cases and non-lesional skin tissue from 16 independent individuals as controls. Results: We identified NMSC-associated STR somatic mutations. These are associated with the dysregulation of DNA damage and repair mechanisms. In artificial intelligence (AI) predictive modeling, these markers could successfully differentiate basal cell carcinoma (BCC) and non-lesional skin tissue. To our knowledge, we present the first study focusing on STR somatic mutations in multiple cancer-related genes in NMSC found only in tumor tissue and not in non-lesional skin tissue. Some of them (*APC*, *BRAF*) are associated with more pronounced dysregulation of relevant gene pathways (hedgehog, Notch signaling, and Wnt signaling). Conclusions: Our findings suggest that this STR somatic mutation status might potentially be used to select BCC patients who could benefit from certain precision therapy including hedgehog inhibitors, gamma-secretase inhibitors, anti-Vasuclar endothelial growth factor (*VEGF*), proteasome inhibitors, and immune check-point inhibitors.

## Simple Summary

In a DNA sequence, there are some regions where a set of DNA bases is repeated right next to others (in tandem), called short tandem repeats (STRs). Sometimes, there are repeats of only one base (e.g., AAAAA or TTTTT). Mutations in such segments are not well studied. This study focused on these repetitive DNA changes in non-melanoma skin cancer (NMSC) across many cancer-related genes. The study identified changes that were only found in the cancerous tissue, not in normal skin. These mutations are associated with significant dysregulations of known cancer-related gene pathways. The findings suggest that these mutations can help identify NMSC patients who might benefit from personalized treatments, like hedgehog (Hh) inhibitors, immune checkpoint inhibitors (ICIs), and other precision therapies. This is the first study to find a new way of identifying and possibly treating skin cancer by looking at specific, previously ignored, repetitive patterns in tumor DNA.

## 1. Introduction

We have previously reported the somatic mutation profile in non-melanoma skin cancer (NMSC) in an arsenic exposed population and shown the associations of single-nucleotide variations (SNVs) and differential gene expression [1]. We also found that somatic mutations in the form of small deletions (DELs) are associated with the dysregulation of important gene pathways [2]. In that study, we observed that many of the significant DELs were detected in simple sequence repeat (SSR) or short tandem repeat (STR) regions—more specifically, in homopolymeric regions. Homopolymeric regions are stretches of DNA where only a single nucleotide is repeated multiple times (e.g., AAAAA or TTTTT). These regions are a subset of STRs but specifically refer to the repetition of only a single nucleotide. STR mutations in homopolymeric regions mainly involve changes in the length due to insertions or deletions (INDELs) of the repeated nucleotide. Traditionally, studies of somatic mutation in cancer typically report SNVs or copy number variations (CNVs), which are mostly detected in the non-homopolymeric regions of tumor DNA. In this study, we have focused on somatic mutations of NMSC detected in homopolymeric regions.

STRs are interchangeably called microsatellites, where each repeat unit is located right next to the other (in tandem), and depending on the number of bases in a “repeat unit”, they are called mono-, di- tri-, tetra-, penta- or hexa-nucleotide repeats. Usually, motifs of up to six bases are called microsatellites. Microsatellites represent three percent of the human genome [3]. Tandem repeats are ubiquitous, unstable, and have historically been designated as nonfunctional “junk DNA” [4]. However, recent studies have suggested that as many as 10% to 20% of eukaryotic genes and promoters harbor an unstable repeat element. Longer microsatellite alleles are more mutagenic and tend to decrease in length, whereas the opposite is seen for shorter alleles [5]. The bulk of simple repeats are embedded in non-coding DNA, either in the intergenic sequence or in introns. A study revealed ~10 million microsatellites across the human genome [6].

Microsatellite instability (MSI) refers to the condition where these repeat sequences become unstable in length due to defects or mutations in mismatch repair (MMR) genes (e.g., MLH1, MSH2, MSH6, and PMS2). This is often associated with certain cancers [7,8,9,10,11,12,13,14,15,16]—mainly colorectal cancer (CRC) [15,16]. For MSI detection in CRC, a panel of mono-nucleotide markers (BAT-25 and BAT-26) and di-nucleotide repeat markers (D2S123, D5S346, and D17S250) are used. Tri-nucleotide and other longer repeats are important in other genetic disorders, such as Huntington’s disease (a CAG tri-nucleotide repeat expansion) and Fragile X syndrome (a CGG tri-nucleotide repeat expansion). MSI markers may be detected in blood or germline DNA if it is inherited, like Lynch syndrome [16], but in sporadic CRC, these are acquired somatic changes and are found in tumor DNA.

The mutation rate of microsatellites is higher than that of other genomic regions due to DNA polymerase slippage during DNA replication and repair [3]. Analysis of mutations in MMR genes showed that somatic SNVs and small INDELs had larger functional impacts than germline mutations and structural variations [6].

MSI is poorly understood in the context of human skin cancers. Researchers showed that well-known detection devices were not indicative of MSI and therefore concluded that MSI is extremely rare in NMSC [17,18]. However, a recent study reports a high-level of MSI in one squamous cell carcinoma (SCC) cell line [19]. In addition, multiple studies confirmed the loss of heterozygosity (LOH) at chromosome 9p.21 region, a locus that may harbor a tumor suppressor gene, using microsatellite markers [20,21].

Very recently, the significance of STRs has been highlighted in the context of human diseases [22,23]. INDELs in such regions have been reported in germline DNA; however, to our knowledge, no human studies have shown the significance of “somatic mutation” in NMSC tissue in homopolymeric regions, which is the simplest form of STR. In this study, we sequenced more than 400 cancer-related genes in NMSC tissues and corresponding blood from the same individuals and non-lesional skin tissue with paired blood. We showed that more than 50% of the somatic mutations found in NMSC tissue are in homopolymeric regions. Among these somatic mutations in STR regions we (a) identified NMSC-associated mutations that were not seen in non-lesional skin tissue; (b) checked if these STR somatic mutations have functional association(s) with the dysregulation of gene pathways; and (c) investigated whether these molecular genomic changes could help in precision medicine in patient selection for targeted therapy if needed.

## 2. Materials and Methods

This study included the first 32 patients developing histopathologically proven NMSC as cases from a large-scale randomized trial in an arsenic-exposed population [24]. Non-lesional, apparently healthy skin tissues surrounding the margins of arsenical keratosis lesions from 16 independent patients served as normal controls. These samples were used in our previous studies reporting SNVs [1] and small DELs [2]. Patient characteristics are in Supplementary Table S1. In this study, we focus on somatic mutations in STR regions only. All tissue samples (NMSC and non-lesional or apparently healthy) were preserved in RNA stabilizing buffer (RNAlater, Thermo Fisher Scientific, Waltham, MA, USA) and stored at −86 °C. DNA and RNA extracted from these ideally preserved tissues were used for sequencing. For both cases and controls, we collected whole blood samples in EDTA tubes from the same individuals, which served as the source of germline DNA for comparison. The sequencing data from tissue DNA was compared against the corresponding germline DNA (blood DNA) from the same patient to identify somatic mutations in each tissue sample. Thus, we sequenced 96 DNA samples (32 NMSC tissue DNA samples and 32 blood DNA samples from the same cases; 16 non-lesional skin tissue DNA samples and 16 blood DNA samples from the same controls). RNA sequencing was performed on 48 RNA samples (32 NMSC and 16 non-lesional skin tissue).

### 2.1. Next Generation Sequencing (NGS)

We sequenced ~1.75 Mb of genomic region per sample, covering mainly the exon regions of 409 cancer-related genes (for complete list: https://support.illumina.com/sequencing/sequencing_kits/ampliseq-for-illumina-comprehensive-cancer-panel/product-compatibility.html, accessed on 11 March 2025) using a commercially available targeted amplicon sequencing-based kit (AmpliSeq for Illumina Comprehensive Cancer Panel, Illumina, San Diego, CA, USA). The primers were designed for 15,992 genomic regions, mainly to capture the coding regions. Intronic or non-coding regions were not the focus of library generation.

### 2.2. Somatic Mutation Detection

The NGS data were processed using CLC Genomic Workbench v25.0 (https://digitalinsights.qiagen.com/; accessed on 1 November 2024) as described previously [1,2]. For somatic mutation detection, paired tissue DNA and blood DNA from the same individual were used as input for the biomedical workflow’s Somatic Cancer Targeted Amplicon Sequencing (TAS) module for tumor–normal pairs. Reads were mapped to hg19, using default parameters. Structural variant caller v1.2 was used. After local re-alignment, low-frequency variant detection v2.5 was used for both the tissue and blood DNA samples. Then, the marginal variants were removed (using Remove Marginal Variant 1.3). In the next step, the variants found in the control (blood) were removed from the variants in the tissue sample. Next, the homozygous reference variants were removed. Finally, the variants were filtered with stringent criteria: minimum variant count = 2, minimum coverage = 30, min frequency = 2%, average quality (Q-score) minimum = 30, mutation call quality minimum = 200 (in Phred scale, meaning probability of error = 1 in 10^20^), and control count = 0. A variant was considered to be present in a homopolymeric region if there were at least four consecutive repeats at that location (string of A’s, T’s, C’s, or G’s).

### 2.3. Transcriptome Wide Gene Expression and Statistical Analysis

We used the gene expression data of these tissue samples from our previous studies [1,2,25]. For transcriptomic data processing, we used Partek Flow (version 10.0) (https://www.partek.com/partek-flow/, accessed on 11 November 2022) using the STAR aligner for alignment, and the final gene count data were expressed as count per million (CPM) reads. Log_2_-transformed CPM data were used for analysis of variance (ANOVA) and gene set ANOVA as described in the previous paper [1,2,25].

### 2.4. Artificial Intelligence (AI) Modeling for Prediction

We used the R Studio (2024.04.2+764) software (https://www.R-project.org/) and the h2o library (https://CRAN.R-project.org/package=h2o, accessed on 26 March 2025) and initialized an h2o.ai cluster. We read in our data frame, which consisted of the top 20 genes with the highest basal cell carcinoma (BCC) associated STR somatic mutation frequencies and divided the data into two sections—a training set (70%) and a validation set (30%). A five-fold cross-validation was used. We used the h2o automatic machine learning (autoML) function, which automates a supervised learning process that trains and tunes a variety of models, such as deeplearing (DL), gradient boosting machine (GBM), distributed random forest (DRF), generalized linear model (GLM), eXtreme gradient boosting (XGBoost), and StackedEnsemble (SE).

## 3. Results

The median number of reads per sample was 12 million, with slight but non-significant variation by the type of DNA sample—a median of 14 million reads for each NMSC sample, 21 million reads for each of the non-lesional skin tissue samples, and 9.6 million reads for each corresponding blood DNA sample (*p* = 0.14, Kruskal–Wallis test; see Supplementary Table S2). By comparing the paired tissue–blood samples (both for NMSC and non-lesional groups) from the same individual, we first identified somatic mutations in 32 NMSC tissues (a total of 5471 incidences) and in 16 non-lesional skin tissues (a total of 2084 incidences). Thus, from 32 NMSC tissue DNA samples and 16 non-lesional skin DNA samples, we identified 7555 incidences of somatic mutations. Interestingly, 4485 (59.4%) were detected in homopolymeric regions. By homopolymeric region, we mean a DNA sequence containing a stretch of the same nucleotide (string of A’s, T’s, C’s, or G’s). A homopolymeric region looks like “AAAAAAAA” or “TTTTTTTT”. These regions are a subset of STRs but specifically refer to the repetition of only a single nucleotide. Therefore, it may be considered the simplest form of a STR or a mononucleotide repeat microsatellite. STR mutations in homopolymeric regions mainly involve changes in the length of these homopolymeric regions due to insertions (INSs) or deletions (DELs) of the repeated nucleotide. In our data, the median length of the homopolymeric region with a mutation was 10 bp. Some of the characteristics of these STR mutations (mutations detected in homopolymeric regions; *n* = 4485) and non-STR somatic mutations (mutations not in homopolymeric regions; *n* = 3070) are presented in Supplementary Table S3. The median variant allele frequency (VAF) in these STRs in homopolymeric regions was ~4%. Among these 4485 STR somatic mutations, 4331 (96.5%) were INDELs. Only a few were SNV events (*n* = 56); the rest were multi-nucleotide variants (MNVs) and substitutions. We restricted our further analyses to these 4331 INDEL events (2043 insertion and 2288 deletion events) in 48 samples (32 NMSC and 16 non-lesional skin tissues) that covered a unique 1151 genomic coordinates in 249 genes.

**Figure 1:**
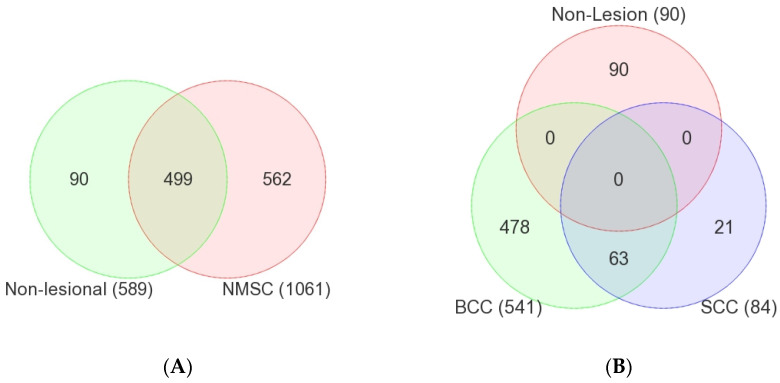
The left Venn Diagram (**A**) presents the overlap of somatic mutations found in non-lesional tissue and non-melanoma skin cancer (NMSC) tissue. The right Venn Diagram (**B**) is similar, showing the overlap of somatic mutations detected in non-lesional, basal cell carcinoma (BCC) and squamous cell carcinoma (SCC) tissue samples.

Figure 1A shows the overlap of the detected STR somatic mutations in NMSC tissues (at 1061 genomic coordinates) and in non-lesional skin tissue (at 589 genomic coordinates). Notably, a large proportion of STR somatic mutation loci in NMSC tissue (499 out of 1061 or 47%) were also common in non-lesional skin tissue. We called these “common STR somatic mutations”. These may be pre-malignant but may not be used as diagnostic markers for NMSC, because these STR mutations are also seen in non-lesion skin tissues. On the other hand, we found STR somatic mutations in 562 genomic coordinates that we found only in NMSC tissue but not in non-lesional skin tissue. We called these “NMSC-associated STR somatic mutations” (see Figure 1B). Similarly, there were STR somatic mutations in 90 genomic coordinates that were “Non-lesional skin associated”.

### 3.1. Common Non-Cancer-Specific STR Somatic Mutations

**Figure 2:**
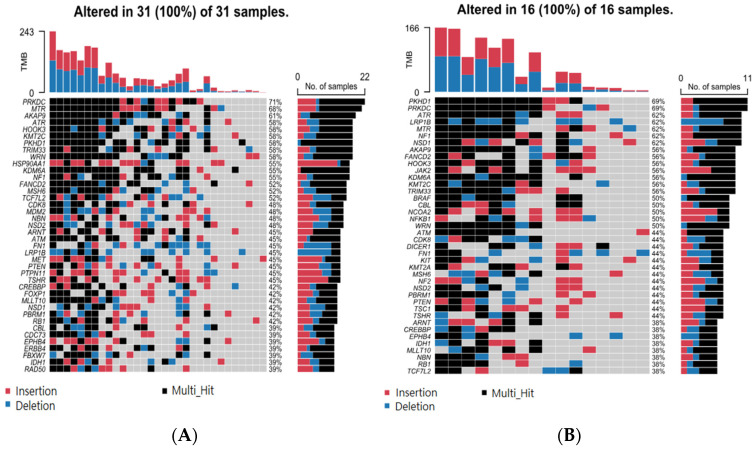
Top 40 genes with non-cancer-specific short tandem repeat (STR) mutations in NMSC tissue (**A**) and top 40 genes harboring non-cancer-specific somatic mutations in non-lesional tissue (**B**). Deletions are in blue, insertions are in red, and multiple hits are in black. Genes are shown in rows, while each column represents an individual patient.

**Figure 3:**
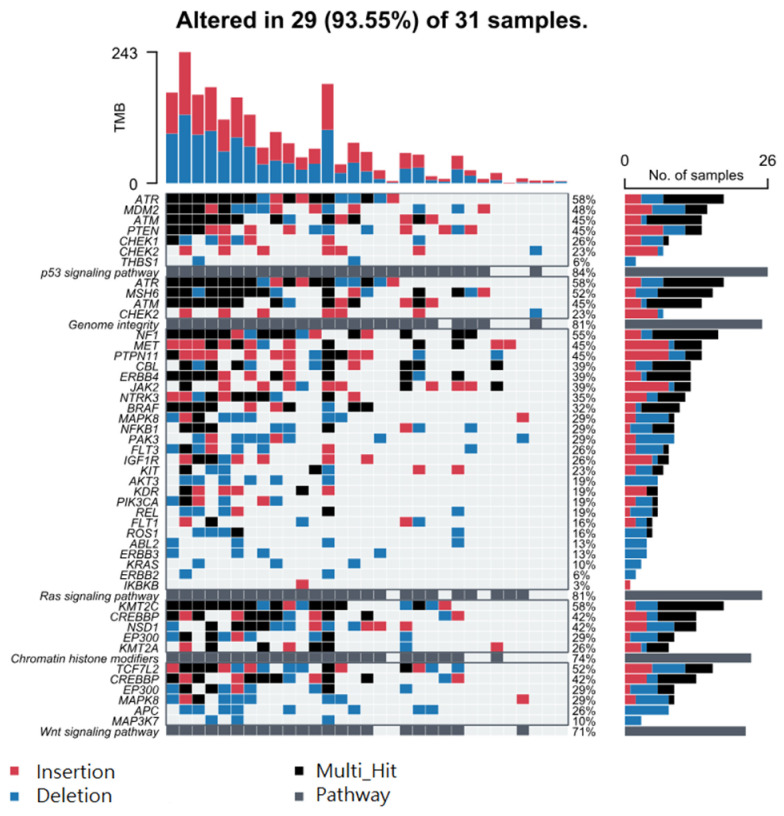
Top 5 pathways harboring non-cancer-specific STR mutations in NMSC tissue. Genes are ranked from highest to lowest frequency in their respective pathway. Deletions are in blue, insertions in red, multiple hits in black, and pathway hits in gray. Pathways and their genes are shown in rows, while each column represents an individual patient.

**Figure 4:**
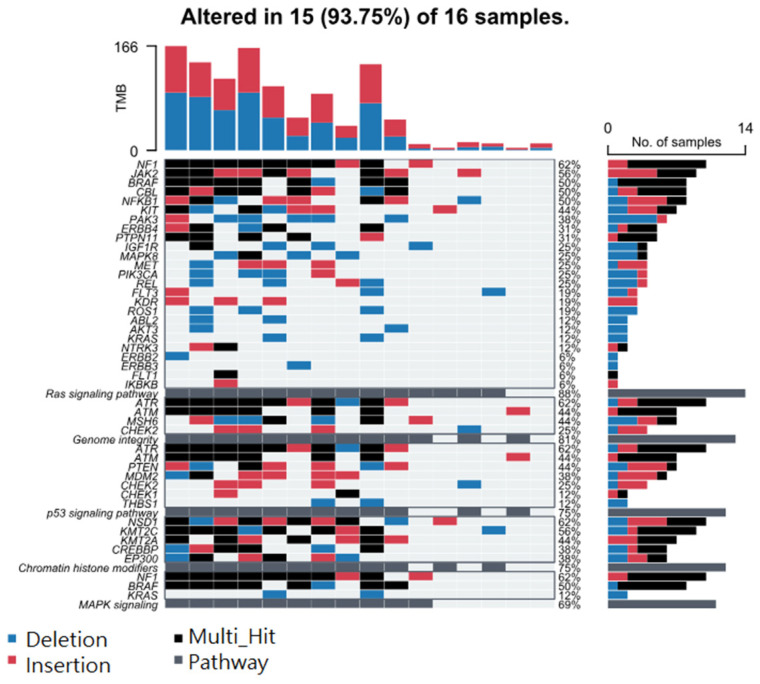
Top 5 pathways harboring non-cancer-specific STR mutations in non-lesional tissue. Genes are ranked from highest to lowest frequency in their respective pathway. Deletions (DELs) are in blue, insertions (INSs) in red, multiple hits in black, and pathway hits in gray. Pathways and their genes are shown in rows, while each column represents an individual patient.

Considering STR somatic mutations at the individual gene level, Figure 2A shows the top 40 genes (by frequency) that harbor the 499 common non-cancer-specific somatic STR mutations in NMSC tissue. Similarly, the top 40 genes that harbor those common STR somatic mutations in non-lesion skin tissues are presented in Figure 2B. We note that 100% of the tissue samples had a STR somatic mutation in at least one of these 40 genes. Considering even a single gene, *PRKDC*, one or more STR somatic mutations were found in ~70% of the NMSC and non-lesional skin tissues. This emphasizes the fact that non-cancer-specific mutations are commonly encountered in skin tissue. When we considered groups of genes (involved in a pathway) that harbor these non-cancer-specific somatic STR mutations (see Figure 3), we found that, for the NMSC samples, 84% of the samples had non-cancer-specific STR somatic mutation in at least one gene of the p53 signaling pathway; 81% of those samples had non-cancer-specific STR somatic mutation in at least one gene associated with genome integrity; and 81% of the samples had non-cancer-specific STR somatic mutation in at least one gene associated with the Ras signaling pathway. A similar proportion of non-lesional skin tissues also harbored non-cancer-specific STR somatic mutations in genes involved in those same pathways (see Figure 4).

### 3.2. NMSC-Associated STR Somatic Mutations

**Figure 5:**
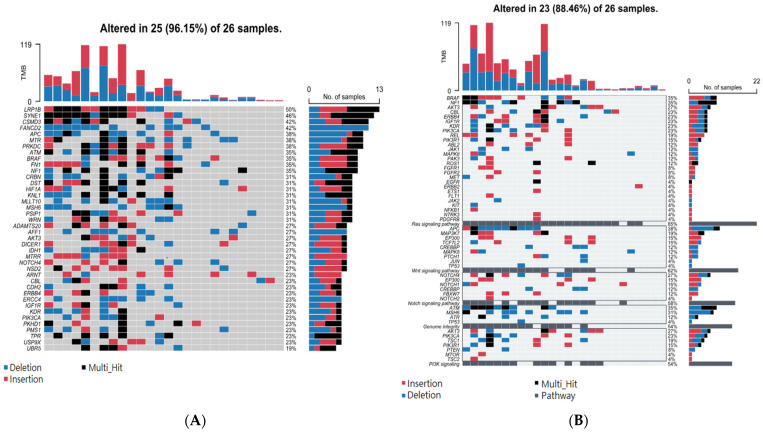
Top 40 genes with BCC-specific STR mutations (**A**) and top 5 pathways harboring BCC-specific somatic mutations (**B**). Genes are ranked from highest to lowest frequency in their respective pathway. DELs are in blue, INS in red, multiple hits in black, and pathway hits in gray. Pathways and their genes are shown in rows, while each column represents an individual patient.

In contrast to the non-cancer-specific STR somatic mutations mentioned above, these NMSC-associated STR somatic mutations were found only in NMSC tissue, not non-lesional skin tissues. The top 40 genes that harbor these NMSC-associated STR somatic mutations are presented in Supplementary Figure S1A. When we considered the groups of genes (involved in a pathway) that harbor these NMSC-associated somatic STR mutations (see Supplementary Figure S1B), we found that 84% of the NMSC samples had cancer-specific STR somatic mutations in at least one gene of the Ras signaling pathway; 56% of the samples had a cancer-specific STR somatic mutation in at least one gene associated with the Wnt signaling pathway; 56% samples had a cancer-specific STR somatic mutation in at least one gene involved in genome integrity; and 50% of the samples had a cancer-specific STR somatic mutation in at least one gene associated with the Notch signaling pathway (see Supplementary Figure S1B). These pathways are biologically relevant to NMSC and are the STR mutations related to genome integrity. The result suggests that non-cancer-specific STR somatic mutations and NMSC-associated STR somatic mutations occur in the same gene or group of genes. Therefore, mere detection of somatic mutation is not enough for diagnostic or therapeutic purposes; rather, detection of NMSC-associated or cancer-specific STR somatic mutations is important. In subgroup analysis, we looked at mutations in BCC (*n* = 26) and SCC (*n* = 6). BCC-associated STR somatic mutation findings at the single-gene level and gene-group level are presented in Figure 5A and Figure 5B, respectively.

### 3.3. Association of BCC-Associated STR Somatic Mutations with MSI-Related Gene Mutation

Considering that these somatic mutations are in homopolymeric regions, we asked if these result from or are associated with mutations in known MSI-related genes responsible for MMR machinery. We had mutation data for *MLH1*, *MSH2*, *MSH6,* and *PMS2* genes for all 26 BCC tissues. The correlations between NMSC-associated somatic mutation status in these four MSI-related genes (0 = no mutation; 1 = mutation; shown in the first four columns of Table 1) and the top 10 BCC-associated STR somatic mutation status (0 = no mutation; 1 = mutation; shown in rows) in these 26 BCC samples are presented in Table 1. Our data suggest a potential link between (a) mutation in *PMS2* with the BCC-associated STR somatic mutations in *LRP1B*, *PRKDC*, *ATM*, and *BRAF* and (b) mutation in *MLH1* and STR somatic mutation in *APC* (significant associations between MMR and STR mutations are highlighted in yellow in Table 1). Table 1 also shows the correlations (or strength of co-occurrence; highlighted in blue) among the STR somatic mutations themselves. We also tested all these samples for the traditional MSI markers (BAT25, BAT26, and CAT25) that are used for colorectal cancer. Only two tumor samples were positive for BAT25 but the corresponding blood samples were also positive, indicating that the BAT25 positivity was not a somatic event in either case; rather, it represented a germline variation.

### 3.4. Association of BCC-Associated STR Somatic Mutations and Differential Gene Expression in BCC Tissue

**Figure 6:**
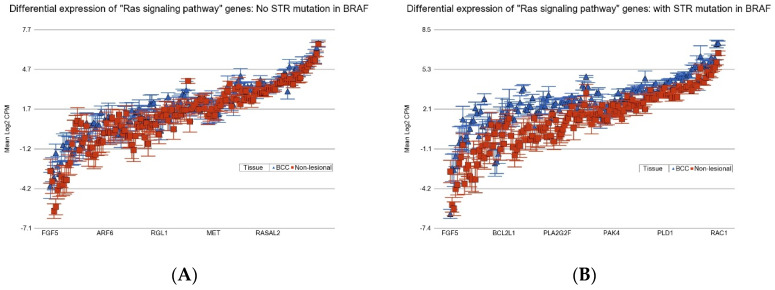

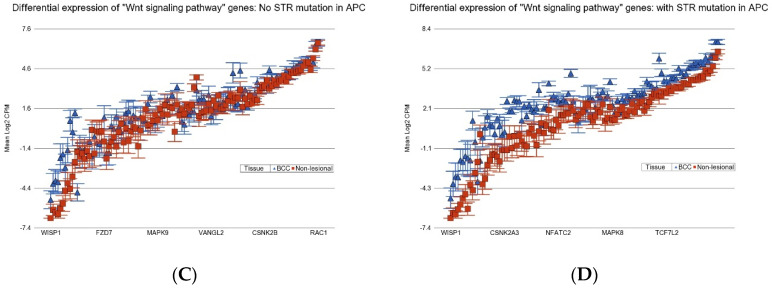
Differential expression “Ras signaling pathway” genes (top panels) and “Wnt signaling pathway” genes (bottom panels) in BCC tissue (in blue) compared to non-lesional tissue (in red). BCC tissues without a STR mutation in *BRAF* are on the top left (**A**) and BCC with STR mutation in *BRAF* are on the top right (**B**). BCC tissues without a STR mutation in *APC* are on the bottom left (**C**) and BCC with STR mutation in *APC* are on the bottom right (**D**). Genes are arranged on the *x*-axis by expression level, and the log2-transformed count per million (CPM) is shown on the *y*-axis. Therefore, a difference of one unit on the *y*-axis represents a 2-fold change. Gene symbols could not be shown for all the genes on the *x*-axis.

In the next step, we asked if the presence of frequently encountered STR somatic mutations had any effect on the differential expression of related gene expression pathways mentioned in the earlier section. Considering the fact that (a) all of the study participants were exposed to arsenic through contaminated drinking water, and (b) in the previous studies, we have shown the interaction of arsenic exposure status and gene expression [2,25], for all the gene set ANOVA models, we entered the arsenics exposure status (0: urinary arsenic-to-creatinine ratio (UACR) ≤ 192 µg/g creatinine; 1: UACR > 192 µg/g; dichotomized by the median for the original study population) [26] along with the mutation status of a given gene we wanted to test. To check if the presence or absence of a STR mutation in a given gene influences the magnitude of the differential expression of a given pathway, we introduced an interaction term Tissue (0: non-lesional; 1: BCC) x STR mutation status (0: no STR mutation; 1: STR mutation present) in the model. If the interaction term *p*-value was significant, that suggested that the magnitudes of differential expression in the presence or absence of a SRT mutation was significantly different. Among the “Ras signaling pathway” genes, *BRAF* was the gene that had a STR mutation in a homopolymeric region most frequently in the population (35% of patients). Compared to the non-lesional skin tissues, in BCC tissues without a STR mutation in *BRAF*, “Ras signaling pathway” genes were overexpressed on average by 1.41-fold (95% CI 1.29–1.54; see Figure 6A), whereas in BCC tissues, with a STR somatic mutation in *BRAF*, those same “Ras signaling” genes were more markedly overexpressed, by 2.47-fold (95% CI 2.22–2.75; see Figure 6B) (ANOVA interaction *p* = 2.29 × 10^−25^). This shows the association of STR somatic mutations in BRAF and the differential gene expression of the “Ras signaling pathway”. Similarly, STR somatic mutations in *APC* were also associated with more marked overexpression of the “Wnt signaling pathway” with a fold change (FC) = 2.66 (95% CI 2.35–3.0) compared to those without *APC* STR somatic mutations, with a FC of 1.56 (95% CI 1.39–1.73) (ANOVA interaction *p* = 6.3 × 10^−17^; see Figure 6C,D, respectively).

### 3.5. Association of BCC-Associated STR Somatic Mutations and Known Cancer-Related Pathways

**Figure 7:**
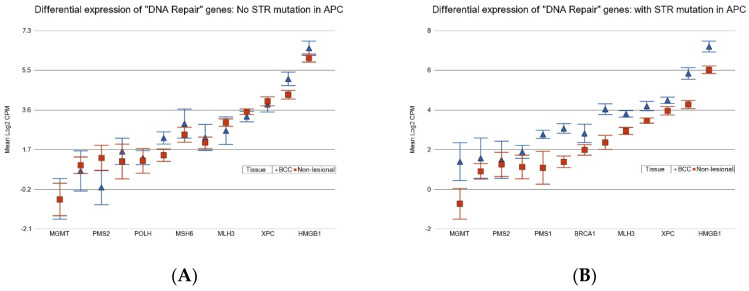
Differential expression “DNA Repair” genes in BCC tissue (in blue) compared to non-lesional tissue (in red). BCC tissues without a STR mutation in *APC* are on the left (**A**) and BCC tissues with a STR mutation in *APC* are on the right (**B**). Genes are arranged on the *x*-axis by expression level, and the log2-transformed gene CPM is shown on the *y*-axis. Gene symbols for all the genes could not be displayed on the *x*-axis.

Considering the different biological processes altered in cancer, we asked if the presence of some of these frequently encountered STR somatic mutations influence the differential expression (BCC tissues vs. non-lesional tissues) of (a) anti-tumor suppressor genes, (b) pro-apoptosis genes, (c) DNA repair genes, (d) tumor suppressor genes, (e) caspase executor genes, (f) caspase initiator genes, and (g) anti-apoptosis genes. In general, one or more of these gene pathways are dysregulated in cancer. The analysis results for BCC-associated STR somatic mutations in *LRP1B*, *SYNE1*, *CSMD3*, *APC*, and *BRAF* are presented in Supplementary Tables S4–S8, respectively. We found that compared to non-lesional tissue, the DNA repair genes were more markedly overexpressed in BCC tissue if the tumor had an STR somatic mutation in *APC* with a FC of 1.97 (95% CI 1.45–2.67, see Table S4) vs. if the BCC tissue did not have an STR somatic mutation in *APC* with a non-significant FC of 1.09 (95% CI −1.19–1.43), (see Figure 7, ANOVA interaction *p* = 0.0002). Similarly, DNA repair genes were also markedly overexpressed in the presence of a BCC-associated STR somatic mutation in BRAF (see Supplementary Table S8).

**Figure 8:**
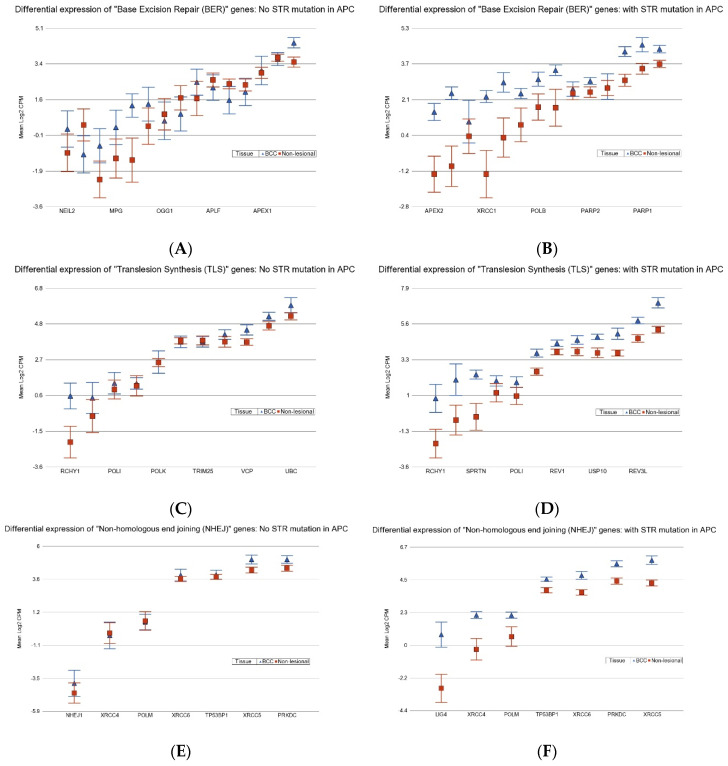
Differential expression of genes related to “base excision repair” (BER) (upper panel), “translesion synthesis” (TLS) (middle panel), and “non-homologous end joining” (NHEJ) (lower panel) in BCC tissue (in blue) compared to non-lesional tissue (in red). BCC tissues without a STR mutation in *APC* are on the left (**A**,**C**,**E**) and BCC with a STR mutation in *APC* are on the right (**B**,**D**,**F**). BER genes were more overexpressed in BCC tissue if the tumor had a STR somatic mutation in *APC* with a fold change (FC) of 2.55 (95% confidence interval (CI) 1.77–3.66) vs. a FC of 1.41 (95% CI 1.03–1.93) (analysis of variance (ANOVA) interaction *p* = 0.002) if the BCC tissue did not have a STR somatic mutation in *APC*. TLS genes were more overexpressed in BCC tissue if the tumor had a STR somatic mutation in *APC* with a FC of 2.56 (95% CI 1.85–3.55) vs. a FC of 1.53 (95% CI 1.15–2.03) (ANOVA interaction *p* = 0.002) if the BCC tissue did not have a STR somatic mutation in *APC*. NHEJ genes more overexpressed in BCC tissue if the tumor had a STR somatic mutation in *APC* with a FC of 3.07 (95% CI 1.99–4.73) vs. a FC of 1.54 (95% CI 1.06–2.25) (ANOVA interaction *p* = 0.002) if the BCC tissue did not have a STR somatic mutation in *APC*. Genes are arranged on the *x*-axis by expression level, and the log_2_-transformed CPM is shown on the *y*-axis. Gene symbols for all the genes could not be shown on the *x*-axis.

Assuming that overexpression of DNA repair genes is the functional response to DNA damage, the result indicates that these STR mutations may be associated with more severe DNA damage and, hence, the DNA repair mechanism. In the next step, we checked the differential expression of genes related to DNA damage pathways (see Supplementary Tables S9 and S10). We found that STR somatic mutations in *APC* were associated with marked overexpression of genes related to pathways such as “base excision repair” (BER) (see Figure 8, upper panel), “translesion synthesis” (TLS) (see Figure 8, middle panel), and “non-homologous end joining” (NHEJ) (see Figure 8, lower panel). Similarly, STR somatic mutations in BRAF were associated with marked overexpression of genes related to “nucleotide excision repair” (NER), BER, and TLS. Our data suggest an association of STR somatic mutation in *APC* and *BRAF* with DNA damage and DNA damage repair process in BCC pathogenesis.

### 3.6. Association of BCC-Associated STR Somatic Mutations and Gene Pathways Known to Be Dysregulated in BCC

For the frequently encountered STR somatic mutations in BCC, we examined interactions with differential expressions of KEGG pathway genes. The detailed results of gene set ANOVA for *LRP1B*, *SYNE1*, *CSMD3*, *APC*, and *BRAF* are in Supplementary Tables S11–S15, respectively. The details include the number of KEGG pathways tested, number of genes tested for a given pathway, name of the pathway, *p*-values for all the variables included in the ANOVA models, FC, and F-ratio. It is interesting to note that the top known dysregulated pathways in BCC, like the hedgehog signaling pathway, basal cell carcinoma pathway, and Notch signaling pathway, had statistically significant interactions with STR somatic mutations in *APC* and *BRAF*. This indicates that the magnitude of differential expressions (BCC vs. non-lesional tissues) was more pronounced when there was a STR somatic mutation in *APC* (see Supplementary Table S14) or *BRAF* (see Supplementary Table S15), suggesting more marked dysregulation in these well-known gene pathways in BCC to be associated with such STR somatic mutations. More importantly, these results are from real clinical samples, not in cell-line or experimental animal models.

### 3.7. Potential Utility of These STR Somatic Mutations in BRAF and APC in Therapeutic Consideration

#### 3.7.1. Hedgehog Inhibitors

**Figure 9:**
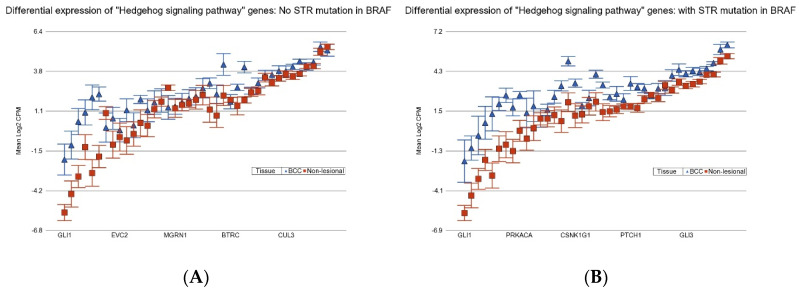
Differential expression “Hedgehog signaling pathway” genes in BCC tissue (in blue) compared to non-lesional tissue (in red). BCC tissues without a STR mutation in *BRAF* are on the left (**A**) and BCC tissues with a STR mutation in *BRAF* are on the right (**B**). “Hedgehog signaling pathway” genes were more overexpressed in BCC tissue if the tumor had a STR somatic mutation in *BRAF* with a FC of 3.08 (95% CI 2.51–3.78) vs. a FC of 2.09 (95% CI 1.76–2.48) (ANOVA interaction *p* = 0.0002) if the BCC tissue did not have a STR somatic mutation in *BRAF*. Genes are arranged on the *x*-axis by expression level, and the log2-transformed CPM is shown on the *y*-axis. Gene symbols for all the genes could not be shown on the *x*-axis.

BCC patients with BCC-associated STR somatic mutations in *BRAF* or *APC* show more marked overexpression of the hedgehog signaling pathway (see Figure 9) and so are more likely to respond to hedgehog (Hh) inhibitors like Vesmodigib or Sonidegib, which are currently used [27,28,29,30,31].

#### 3.7.2. Gamma-Secretase Inhibitors

**Figure 10:**
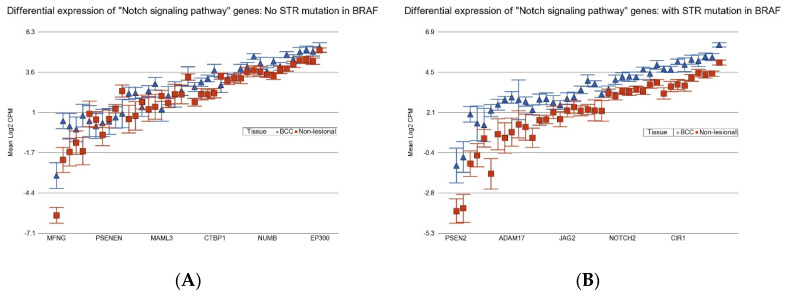
Differential expression “Notch signaling pathway” genes in BCC tissue (in blue) compared to non-lesional tissue (in red). BCC tissues without a STR mutation in *BRAF* are on the left (**A**) and BCC tissues with a STR mutation in *BRAF* are on the right (**B**). “Notch signaling pathway” genes were more overexpressed in BCC tissue if the tumor had a STR somatic mutation in *BRAF* with a FC of 2.55 (95% CI 2.12–3.07) vs. a FC of 1.43 (95% CI 1.22–1.67) (ANOVA interaction *p* = 5 × 10^−10^) if the BCC tissue did not have a STR somatic mutation in *BRAF*. Genes are arranged on the *x*-axis by expression level, and the log_2_-transformed gene CPM is shown on the *y*-axis. Gene symbols for all the genes could not be displayed on the *x*-axis.

BCC patients with BCC-associated STR somatic mutations in *BRAF* or *APC* show more marked overexpression of the Notch signaling pathway (see Figure 10), and so they may be better candidates for the potential use of gamma-secretase inhibitors (GCIs) [32] or antibodies against Notch receptor or ligands [33].

#### 3.7.3. Anti-Vascular Endothelial Growth Factor (VEGF) Therapy

**Figure 11:**
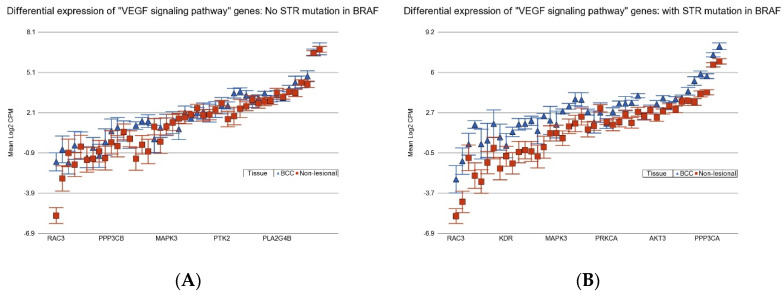
Differential expression “Vascular endothelial growth factor (VEGF) signaling pathway” genes in BCC tissue (in blue) compared to non-lesional tissue (in red). BCC tissues without a STR mutation in *BRAF* are on the left (**A**) and BCC tissues with a STR mutation in *BRAF* are on the right (**B**). “VEGF signaling pathway” genes were more overexpressed in BCC tissue if the tumor had a STR somatic mutation in *BRAF* with a FC of 2.58 (95% CI 2.11–3.14) vs. a FC of 1.43 (95% CI 1.21–1.69) (ANOVA interaction *p* = 5 × 10^−9^) if the BCC tissue did not have a STR somatic mutation in *BRAF*. Genes are arranged on the *x*-axis by expression level, and the log2-transformed gene CPM is shown on the *y*-axis. Gene symbols for all the genes could not be displayed on the *x*-axis.

BCC patients with BCC-associated STR somatic mutations in *BRAF* or *APC* show more marked overexpression of the VEGF signaling pathway (see Figure 11), and so they may be better candidates for potential use of drugs targeting VEGF like bevacizumab, ranibizumab, aflivercept, ramucirumab, or multi-kinase inhibitors like sorafenib or sunitinib.

#### 3.7.4. Proteasome Inhibitors

**Figure 12:**
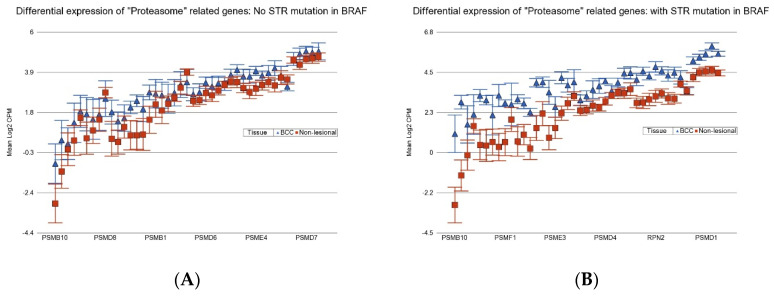
Differential expression “Proteasome” genes in BCC tissue (in blue) compared to Non-lesional tissue (in red). BCC tissues without a STR mutation in *BRAF* are on the left (**A**) and BCC tissues with STR mutation in *BRAF* are on the right (**B**). Proteasome genes were more overexpressed in BCC tissue if the tumor had a STR somatic mutation in *BRAF* with a FC of 2.79 (95% CI 2.33–3.32) vs. a FC of 1.48 (95% CI 1.28–1.72) (ANOVA interaction *p* = 1.87 × 10^−12^) if the BCC tissue did not have a STR somatic mutation in *BRAF*. Genes are arranged on the *x*-axis by expression level, and the log2-transformed gene CPM is shown on the *y*-axis. Gene symbols for all the genes could not be displayed on the *x*-axis.

Other potential targets may be proteasome inhibitors. BCC patients with BCC-associated STR somatic mutations in *BRAF* or *APC* show more marked overexpression of proteasome-related genes (see Figure 12), and so they may be better candidates for the potential use of drugs like bortezomib, carfilzomib, or ixazomib.

#### 3.7.5. Immune Checkpoint Inhibitors (ICIs)

**Figure 13:**
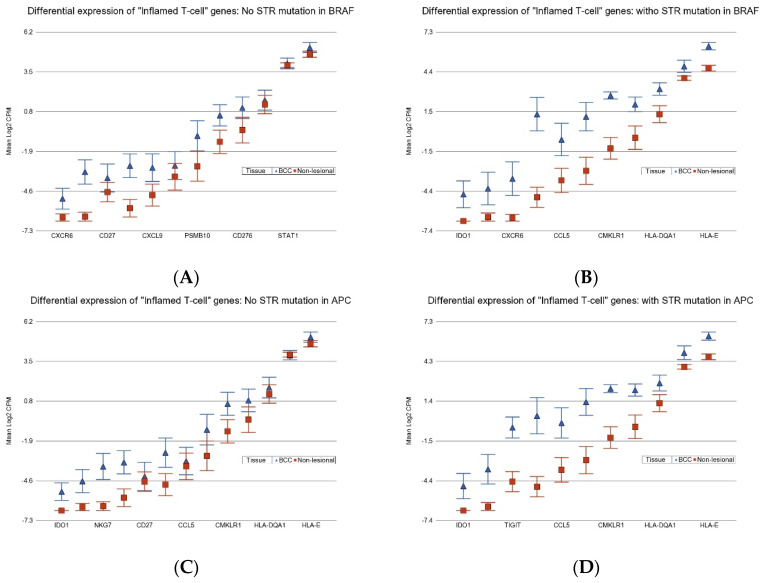
Differential expression “Inflamed T-cell” genes in BCC tissue (in blue) compared to Non-lesional tissue (in red). BCC tissues without a STR mutation in *BRAF* are on the upper left (**A**) and BCC tissues with STR mutation in *BRAF* are on the upper right (**B**). BCC tissues without a STR mutation in *APC* are on the lower left (**C**) and BCC tissues with STR mutation in *APC* are on the lower right (**D**). Genes are arranged on the *x*-axis by expression level, and the log2-transformed gene CPM is shown on the *y*-axis. Gene symbols for all the genes could not be displayed on the *x*-axis.

Our data also suggest that BCC patients with a STR somatic mutation in *BRAF* showed marked overexpression of “inflamed T-cell”-related genes with a FC of 6.1 (95% CI 3.8–9.6) compared to those who did not have a STR somatic mutation in *BRAF* with a FC of 2.3 (95% CI 1.6–3.4) (see the upper panel of Figure 13; ANOVA interaction *p* = 4.21 × 10^−5^). In the same way, BCC patients with a STR somatic mutation in *APC* also showed marked overexpression of “inflamed T-cell”-related genes with a FC of 7.1 (95% CI 4.5–11.2) compared to those who did not have a STR somatic mutation in *APC* with a FC of 4.9 (95% CI 3.3–7.4) (see the lower panel of Figure 13; ANOVA interaction *p* = 0.0006). Overexpression of “inflamed T-cell” genes is a known marker for the potential of a good response to ICI. In this line, our data suggest that the presence of STR somatic mutations in *BRAF* or *APC* may be used to select BCC patients who may benefit from ICI therapy.

### 3.8. Predictive Modeling Using AI

**Figure 14:**
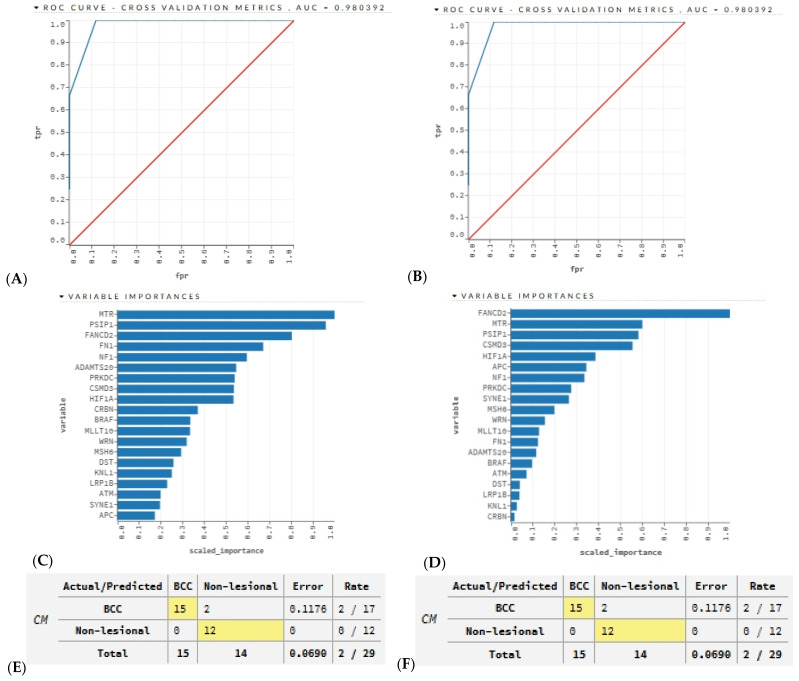
The top panel shows the receiver operating characteristic (ROC) curve cross-validation metrics of the deep learning (DL) model (**A**) and the gradient boosting machine (GBM) model (**B**) and their area under curve (AUC) value based on 20 genes with the highest mutation frequencies in our patient population. The true positive rate is on the *y*-axis, while the false positive rate is on the *x*-axis. The red line represents an AUC of 0.5; where the false positive rate is equal to the true positive rate. The blue line represents the true AUC of the model. The lower panel shows bar graphs of the variable importances of the top 20 genes for the DL model (**C**) and the GBM model (**D**). The *y*-axis lists the variable, while the *x*-axis lists each variable’s scaled importance on a scale from zero to one. The confusion matrices of the DL model (**E**) and the GBM model (**F**) are shown in the bottom row. Cases highlighted in yellow are predicted correctly, whereas cases not highlighted are incorrect. The error percentage is shown as a decimal and the error rate is shown as a fraction. The confusion matrix is based on the 70% samples (*n* = 29 out of 42) that were selected for the training set, and that set was used for 5-fold cross-validation.

Using AI models, we tested if the BCC-associated STR somatic mutation status of the top 20 or the top 10 frequently mutated genes could successfully identify the BCC tissues and the non-lesional skin tissues. We created a training frame and a validation frame, consisting of 70% and 30% of the original data frame, respectively. Next, we used the autoML function with our training and validation frames to produce various models. We used five-fold cross-validation. Overall, the function generated 627 models, and finally, we ranked these models according to the cross-validation receiver operating characteristic (ROC) area under curve (AUC). The top ranked DL model and top ranked GBM model showed the exact same AUC (0.98), which may be considered excellent [34]. The ROC curves from the DL and GBM models are shown in the top panel of Figure 14. The variable importance from these models is shown in the middle panel of Figure 14. Although the variable importance is different in ranking in the DL and GBM models, both had similar performance metrics. The confusion matrices for the DL and GBM models are shown in the lower panel of Figure 14. The accuracy (93%), sensitivity (88%), specificity (100%), positive predictive value (100%), and negative predictive value (85%) calculated from the matrices were promising. The parameters used by AutoML for the DL and GBM models are also shown in Supplementary Files S1 and S2. When we used only 10 genes, the performance did not decline much (AUC was 0.96; see Supplementary Figure S2). We acknowledge the limitation of our small sample size. However, in the future, we plan to deploy these AI models in large testing sets to confirm their performance.

## 4. Discussion

Our study was not designed to specifically interrogate somatic mutation in homopolymeric or STR regions in NMSC. We used a commercially available comprehensive cancer panel of 409 cancer-related genes primarily targeting coding regions. Therefore, we have little coverage on intergenic or intronic regions.

Interestingly, more than 50% of the somatic mutations detected in skin samples (both NMSC and non-lesional) were in homopolymeric regions. It is difficult to sequence homopolymeric regions in different platforms. With respect to the reliability of the mutation results, we feel confident for several reasons. First, acknowledging the possibility of a sequencing error for such a region, we intentionally used very stringent criteria for mutation detection. Second, we used tissue; blood pairs from the same individuals to detect somatic mutations. Low-frequency variants were detected in both tissue and blood DNA against the reference genome, and then the variants detected in blood DNA were removed from the variants detected in skin tissue. Therefore, it is less likely that the sequencing error would occur, mainly in skin tissue samples. Third, a study shows that the error in sequencing in such regions was the lowest with the Illumina platform, which was used to sequence our study samples [35]. Fourth, transcriptome-wide gene expression showed functional relevance in the sense that we observed biologically meaningful gene pathway dysregulation that is expected in NMSC. Fifth, the AI prediction models for classification (BCC vs. non-lesional skin) based on these STR somatic mutation status also suggested that these genomic features could differentiate BCC from non-lesional skin tissue.

Our study detected a large number of the same somatic mutations in non-lesional skin tissue as were also seen in NMSC tissue. Even in a single gene, somatic mutations occurred at multiple loci, some of which developed in both non-lesional and NMSC tissue, and a fraction of them were seen only in NMSC tissue. Therefore, mere detection of somatic mutation even in a known cancer-related gene may not suffice for diagnostic or therapeutic purposes. Rather, identifying and detecting NMSC-associated (or cancer-specific) somatic mutations is important. This study shows that many such NMSC-associated or BCC-associated somatic mutations occur in homopolymeric regions. Homopolymeric regions represent the simplest form of STRs. In a recent study, mutations in such STR regions have been seen in colorectal carcinoma [13]. It may be mentioned that CRC is known to have MSI [12]. Findings from earlier studies related to STR mutation and MSI in colorectal cancer [7,11,12,13], LOH, and other genomic markers in skin cancer [8,10,18,20,21,27,31,36,37,38] are shown in Supplementary Table S16. In general, MSI is rare in NMSC. To our knowledge, we present extensive data on STR somatic mutation in NMSC for the first time.

We identified BCC-associated STR somatic mutations in multiple cancer-related genes. More than 90% of the samples had at least one STR somatic mutation in at least one of the top 40 frequently mutated genes. Some of the top frequently mutated genes harbored STR somatic mutations in 35% to 50% of the cases. We could not determine if these STR mutations were due to the somatic mutation/defect in the MMR gene(s). However, somatic mutation in the MSI-related MMR gene *PMS2* was moderately associated with STR somatic mutation in *BRAF, ATM,* and *PRKDC*, while somatic mutation in the MSI-related MMR gene *MLH1* was moderately associated with STR somatic mutation in *APC*. We also found that these STR somatic mutations were associated with DNA damage and specific DNA repair pathway genes (e.g., NER, BER, TLS, and NHEJ).

Regarding the functional implications of these BCC-associated STR somatic mutations in the top 10 genes, our data suggested that STR mutations in at least *BRAF* and *APC* significantly influenced the magnitude of dysregulation of many gene pathways known to be involved in BCC (e.g., the hedgehog signaling pathway, Notch signaling pathway, and basal cell carcinoma pathway) in a way that the dysregulation of the pathway(s) was more pronounced in patients who had STR somatic mutation(s). STR somatic mutation of other genes had a weaker or less pronounced effect on the dysregulation of known BCC-related gene pathways. While we did not conduct any mechanistic studies, possible mechanisms were reviewed by Gemayel R et al. [4]. We agree that it remains to be determined whether these STR somatic mutations cause differential gene expression or are linked to expression-determining loci in future studies.

AI predictive modeling also showed that the BCC-associated STR somatic mutations we detected in this study were able to correctly separate BCC tissues from non-lesional skin tissue. This also emphasizes the fact that these STR somatic mutations are strong molecular features of BCC. In the future, a larger study may confirm this AI finding.

Regarding the potential practical application of the current findings in precision medicine, our data suggest that, based on the STR somatic mutation status of *BRAF* and *APC* in the BCC tumor tissue, it may be possible to select a group of patients who may respond well to some of the targeted therapy. All of the patients included in this study were treated with surgical excision and did not require additional targeted therapy. However, it may be mentioned that Hh inhibitors like vesmodigib and sonidegib have been used in advanced or metastatic BCC [27,31]. Our data may suggest that patients with BCC-associated STR somatic mutation in *BRAF* may potentially benefit more from such therapy, as they have more pronounced over-expression of the hedgehog signaling pathway. Similarly, GSIs targeting the Notch signaling pathway and anti-VEGF therapy may produce a better response in patients with STR somatic mutation in *BRAF*. Proteasome inhibitors are another class of medications used to treat hematologic malignancy. We are not aware of any studies using such therapy in NMSC. Our molecular data suggest that perhaps BCC patients with STR somatic mutation in *BRAF* may be better candidates for such an approach. ICIs are now widely used for multiple malignancies; our study presents molecular evidence suggesting that BCC patients with STR somatic mutation in *BRAF*, *APC*, or both may better respond to ICI therapy.

Regarding future research perspectives from a translational standpoint, we must first admit that at least for NMSC, these tissue-based genomic markers are not going to help in diagnosis. Histopathology from suspected skin lesions would remain the main avenue for diagnosis. However, these genomic markers may help with precision medicine. Second, the significance of STR somatic mutation may open up the potential of diagnostic research using such STR mutations in cell-free DNA for internal organ malignancy. Third, the detection of these genomic markers in cell free-DNA during post-surgical follow-up can help detect detecting relapses as well as give guidance for targeted therapy.

## 5. Conclusions

Our study identifies STR somatic mutations in multiple cancer-related genes in NMSC that are found only in tumor tissue and not in non-lesional skin tissue. Some of them (*APC* and *BRAF*) are associated with more pronounced dysregulation of relevant gene pathways (hedgehog, Notch signaling, Wnt signaling). Findings also suggest that this STR somatic mutation status might potentially be used to select BCC patients who could benefit from certain precision therapy, including hedgehog inhibitors, GSIs, anti-VEGF, proteasome inhibitors, and ICIs.

## Figures and Tables

**Table 1:** Correlations between STR insertions and deletions (INDELs) and mismatch repair (MMR) genes.

Correlations														
		NMSC_asso_MLH1_mut	NMSC_asso_MSH2_mut	NMSC_asso_MSH6_mut	NMSC_asso_PMS2_mut	LRP1B_STR_BCC	SYNE1_STR_BCC	CSMD3_STR_BCC	FANCD2_STR_BCC	MTR_STR_BCC	PRKDC_STR_BCC	APC_STR_BCC	ATM_STR_BCC	NF1_STR_BCC
LRP1B_STR_BCC	Pearson Correlation	0.081	0.200	0.234	0.426 *									
	Sig. (2-tailed)	0.695	0.327	0.251	0.030									
SYNE1_STR_BCC	Pearson Correlation	0.299	0.216	0.300	0.247	0.617 **								
	Sig. (2-tailed)	0.137	0.289	0.136	0.224	0.001								
CSMD3_STR_BCC	Pearson Correlation	0.031	0.234	0.055	0.066	0.389 *	0.456 *							
	Sig. (2-tailed)	0.879	0.251	0.791	0.747	0.049	0.019							
FANCD2_STR_BCC	Pearson Correlation	0.195	0.234	0.370	0.066	0.389 *	0.613 **	0.370						
	Sig. (2-tailed)	0.340	0.251	0.063	0.747	0.049	0.001	0.063						
MTR_STR_BCC	Pearson Correlation	0.089	−0.158	0.283	0.101	0.158	0.220	0.123	0.123					
	Sig. (2-tailed)	0.664	0.440	0.161	0.623	0.440	0.281	0.549	0.549					
PRKDC_STR_BCC	Pearson Correlation	0.089	0.253	0.123	0.539 **	0.474 *	0.378	0.443 *	0.283	0.513 **				
	Sig. (2-tailed)	0.664	0.212	0.549	0.004	0.014	0.057	0.023	0.161	0.007				
APC_STR_BCC	Pearson Correlation	0.422 *	−0.158	0.123	0.320	0.474 *	0.537 **	0.443 *	0.283	0.350	0.350			
	Sig. (2-tailed)	0.032	0.440	0.549	0.111	0.014	0.005	0.023	0.161	0.080	0.080			
ATM_STR_BCC	Pearson Correlation	0.320	0.275	0.195	0.586 **	0.566 **	0.624 **	0.522 **	0.359	0.256	0.754 **	0.588 **		
	Sig. (2-tailed)	0.111	0.174	0.340	0.002	0.003	0.001	0.006	0.072	0.207	0.000	0.002		
NF1_STR_BCC	Pearson Correlation	0.150	0.275	0.359	0.138	0.243	0.299	0.359	0.195	−0.077	0.256	0.256	0.490 *	
	Sig. (2-tailed)	0.464	0.174	0.072	0.502	0.233	0.137	0.072	0.340	0.710	0.207	0.207	0.011	
BRAF_STR_BCC	Pearson Correlation	−0.020	0.275	0.195	0.586 **	0.243	0.299	0.031	0.031	0.089	0.422 *	0.422 *	0.490 *	0.490 *
	Sig. (2-tailed)	0.924	0.174	0.340	0.002	0.233	0.137	0.879	0.879	0.664	0.032	0.032	0.011	0.011

** Correlation is significant at the 0.01 level (2-tailed); * Correlation is significant at the 0.05 level (2-tailed).

## Data Availability

All the supporting data are presented in the tables presented in the main manuscript and Supplementary Materials.

## Supplementary Materials

The following supporting information can be downloaded at: https://www.mdpi.com/article/10.3390/cancers17101669/s1, Figure S1: Top 40 genes harboring NMSC-specific STR INDELS; Figure S2: Cross-validation metrics of AI models based on top 10 genes only; Table S1: Patient Characteristics; Table S2: Sequence Reads by sample type; Table S3: Variants_by_genomic regions; Table S4: CancerRelatedGenes_interact_STR LRP1B; Table S5: CancerRelatedGenes_interact_STR CSMD3; Table S6: CancerRelatedGenes_interact_STR SYNE1; Table S7: CancerRelatedGenes_interact_STR APC; Table S8: CancerRelatedGenes_interact_STR BRAF; Table S9: DNA Damage Pathway Genes_adjUACR_interact_STR APC; Table S10: DNA Damage Pathway Genes_adjUACR_interact_STR BRAF; Table S11: Geneset ANOVA_KEGG_interact_STR LRP1B; Table S12: Geneset ANOVA_KEGG_interact_STR SYNE1; Table S13: Geneset ANOVA_KEGG_interact_STR LRP1B; Table S14: Geneset ANOVA_KEGG_interact_STR APC; Table S15: Geneset ANOVA_KEGG_interact_STR BRAF; Table S16: Review earlier studies; Supplementary File S1: model_DL_AutoML_model153_20Gene_Info; Supplementary File S2: model_GBM_AutoML_model154_20Gene_Info. References [4,6,7,8,9,10,11,12,13,14,18,19,20,21,27,31,32,33,36,37,38,39,40,41,42,43,44,45,46,47,48,49,50] are cited in the Supplementary Materials.

## Abbreviations

The following abbreviations are used in this manuscript:

NMSC	Non-melanoma skin cancer
As	Arsenic
SNV	Single-nucleotide variation
DEL	Small deletion
SSR	Simple sequence repeat
STR	Short tandem repeat
INDEL	Insertion or deletion
CNV	Copy number variation
DNA	Deoxyribonucleic acid
MSI	Microsatellite instability
MMR	Mismatch repair
CRC	Colorectal cancer
LOH	Loss of heterozygosity
SCC	Squamous cell carcinoma
RNA	Ribonucleic acid
NGS	Next-generation sequencing
EDTA	Ethylenediaminetetraacetic acid
Mb	Megabase
TAS	Targeted amplicon sequencing
AI	Artificial intelligence
DL	Deep learning
GBM	Gradient boosting machine
DRF	Distributed random forest
GLM	Generalized linear model
XGBoost	eXtreme gradient boosting
SE	Stacked ensemble
autoML	Automated machine learning
VAF	Variant allele frequency
MNV	Multi-nucleotide variant
BCC	Basal cell carcinoma
UACR	Urine albumin–creatinine ratio
CPM	Count per million
BER	Base excision repair
TLS	Translesion synthesis
NHEJ	Non-homologous end joining
Hh	Hedgehog
AUC	Area under curve
ROC	Receiver operating characteristic
FC	Fold change
VEGF	Vascular endothelial growth factor
ANOVA	Analysis of variance
GCI	Gamma-secretase inhibitor
ICI	Immune checkpoint inhibitor
