# Supplementary material for: Short Tandem Repeat (STR) Somatic Mutation in Non-Melanoma Skin Cancer (NMSC): Association with Transcriptomic Profile and Potential Implications for Therapy
Source: Cancers (Basel). 2025 May 15;17(10):1669. doi: 10.3390/cancers17101669 (PMC12110349; doi:10.3390/cancers17101669)
Supplement: Supplementary file 1 [file cancers-17-01669-s001.zip › Supplementary Figure S1.pdf]

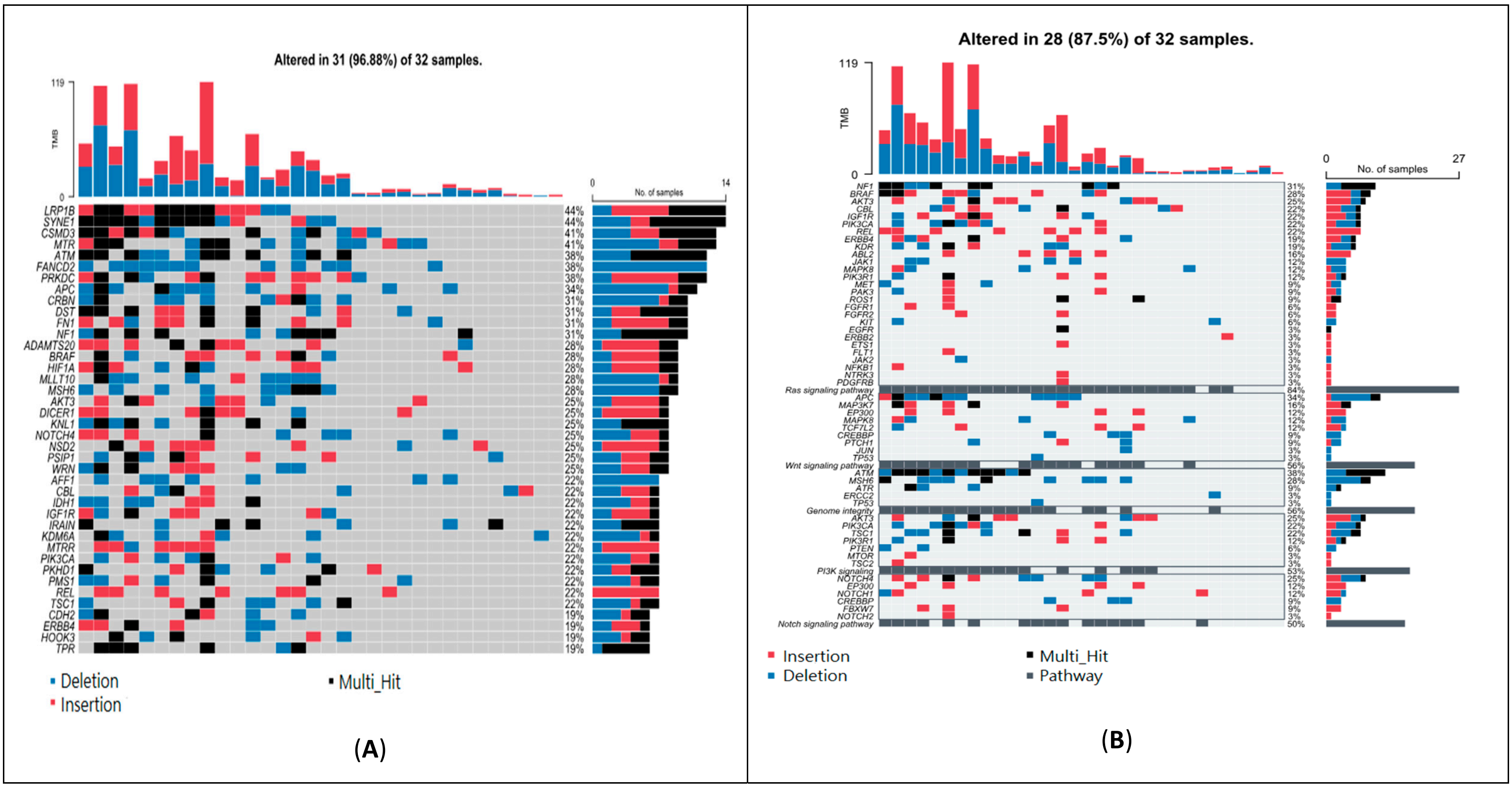

Figure S1. Top 40 genes harboring NMSC-specific STR INDELS (**A**). Top 5 pathways harboring NMSC-specific STR mutations (**B**). Deletions are in blue, insertions are in red, multiple hits are in black, and pathway hits are grey. Pathways and genes are shown in rows, while each column represents an individual patient.
