# Supplementary material for: Short Tandem Repeat (STR) Somatic Mutation in Non-Melanoma Skin Cancer (NMSC): Association with Transcriptomic Profile and Potential Implications for Therapy
Source: Cancers (Basel). 2025 May 15;17(10):1669. doi: 10.3390/cancers17101669 (PMC12110349; doi:10.3390/cancers17101669)
Supplement: Supplementary file 1 [file cancers-17-01669-s001.zip › Supplementary Figure S2.pdf]

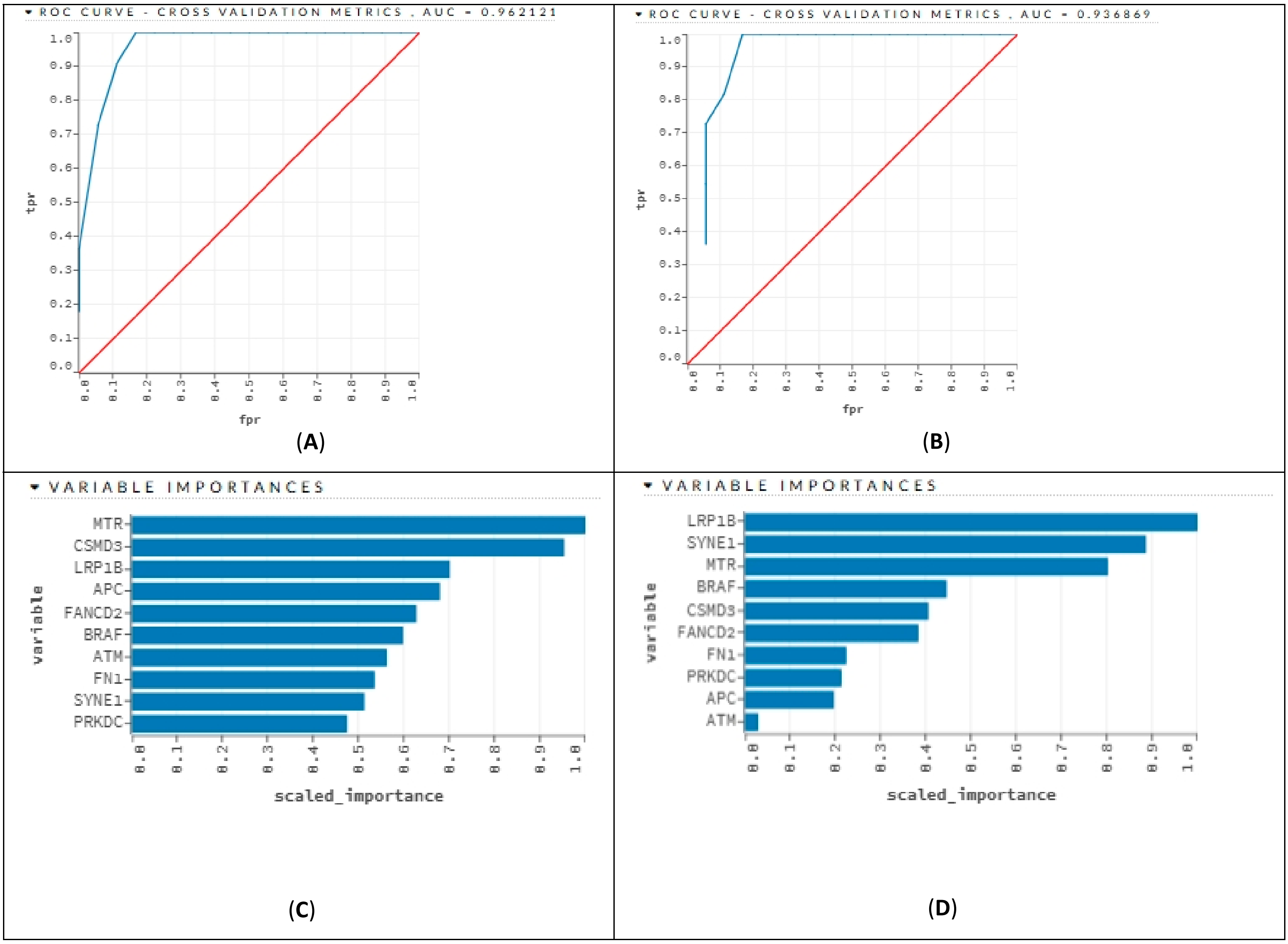

Figure S2. The top panel shows the ROC curve cross validation metrics of the DL model (**A**) and the GBM model (**B**) and their AUC value based on 10 genes with the highest mutation frequencies in our patient population. True positive rate is on the *y*-axis, while the false positive rate is on the *x*-axis. The lower panel shows bar graphs of the variable importances of the top 10 genes for the DL model (**C**) and the GBM model (**D**). The *y*-axis lists the variable, while the *x*-axis lists each variables scaled importance on a scale from zero to one.
